# Sexual reproduction in the Caribbean coral genus *Isophyllia* (Scleractinia: Mussidae)

**DOI:** 10.7717/peerj.2665

**Published:** 2016-11-10

**Authors:** Derek Soto, Ernesto Weil

**Affiliations:** Department of Marine Science, Universidad de Puerto Rico, Recinto de Mayagüez, Mayagüez, Puerto Rico, United States

**Keywords:** Caribbean, Mussidae, Coral reproduction, Hermaphroditic, Brooder

## Abstract

The sexual pattern, reproductive mode, and timing of reproduction of *Isophyllia sinuosa* and *Isophyllia rigida*, two Caribbean Mussids, were assessed by histological analysis of specimens collected monthly during 2000–2001. Both species are simultaneous hermaphroditic brooders characterized by a single annual gametogenetic cycle. Spermatocytes and oocytes of different stages were found to develop within the same mesentery indicating sequential maturation for extended planulation. Oogenesis took place during May through April in *I. sinuosa* and from August through June in *I. rigida*. Oocytes began development 7–8 months prior to spermaries but both sexes matured simultaneously. Zooxanthellate planulae were observed in *I. sinuosa* during April and in *I. rigida* from June through September. Higher polyp and mesenterial fecundity were found in *I. rigida* compared to *I. sinuosa*. Larger oocyte sizes were found in *I. sinuosa* than in *I. rigida*, however larger planula sizes were found in *I. rigida*. Hermaphroditism is the exclusive sexual pattern within the Mussidae while brooding has been documented within the related genera *Mussa*, *Scolymia* and *Mycetophyllia*. This study represents the first description of the sexual characteristics of *I. rigida* and provides an updated description of *I. sinuosa*.

## Introduction

Reproduction in corals consists of a sequence of events which include: gametogenesis, spawning (broadcasters), fertilization, embryogenesis, planulation (brooders), dispersal, settlement and recruitment (Harrison & Wallace, 1990). The success of the reproductive effort is determined largely by the timing, duration, frequency and intensity of the aforementioned events (Babcock et al., 1986). In corals, sexual pattern, mode of reproduction, fertilization, larval dispersal, recruitment and survivorship are key components in determining evolutionary fitness (Szmant, 1986; Edmunds, 2005; Vermeij, 2006; Weil, Croquer & Urreiztieta, 2009; Pinzon & Weil, 2011) which is defined as the product of sexual output (fecundity) and survivorship (Metz, Nisbet & Geritz, 1992). Consequently, the ability of coral species to adapt to modern-day environmental pressures depends greatly on the ability of species to reproduce effectively.

**Figure 1:**
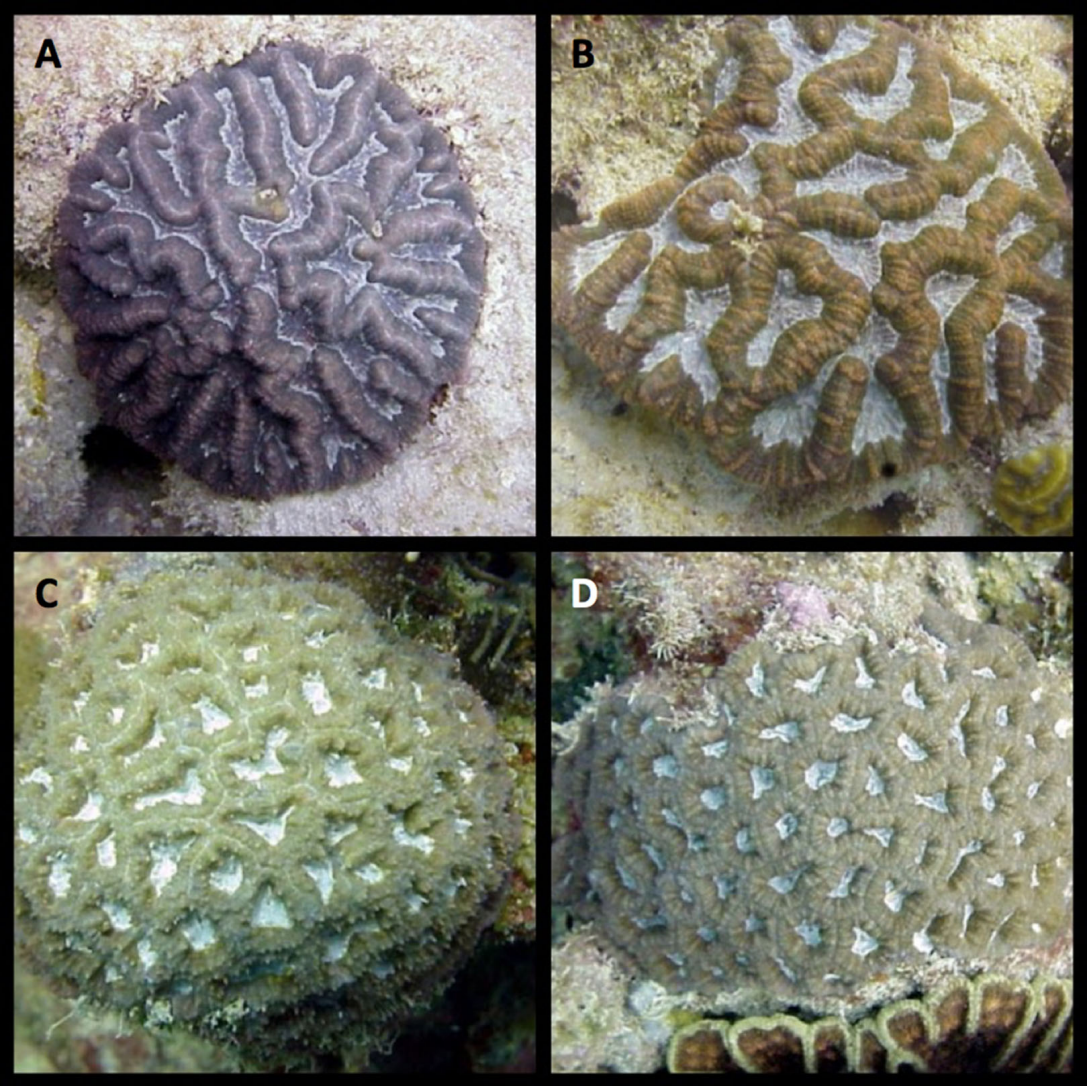
Plate showing study corals. (A, B) *Isophyllastrea rigida* (C, D) *Isophyllia sinuosa*. Photos by Ernesto Weil.

The reproductive characteristics of some scleractinian groups have been more thoroughly studied than others; however, little is known about the reproductive patterns of many Caribbean coral species and some of the available information is conflictive or incomplete (Fadlallah, 1983; Harrison & Wallace, 1990; Harrison, 2011; Weil & Vargas, 2010; Pinzon & Weil, 2011). Of the approximately 60 Caribbean zooxanthellate coral species reported, thorough descriptions of their reproductive characteristics and cycles are available for 19 species; many other studies available provide partial or conflicting results (Weil, 2003; Weil & Vargas, 2010; Harrison, 2011). Reproductive studies of the sexual patterns of *I. sinuosa* were among the first studies of such nature performed in the Caribbean (Duerden, 1902). These were limited to histological observations of oocytes in a few colonies of *I. sinuosa* (Figs. 1A and 1B), therefore, the species is classified as gonochoric. This characterization contrasts with the reproductive mode of other studied Mussids which are classified as hermaphroditic. Currently, there is no information available on the reproductive biology of *I. rigida* (Figs. 1C and 1D).

This study characterizes the reproductive biology of *I. rigida* and *I. sinuosa* in terms of sexual pattern, mode of development, gametogenic cycles, and fecundity. These fundamental aspects of the physiology of this taxa are understudied. Knowledge of the reproductive biology and ecology of coral species is important for the interpretation of their population and ecological dynamics, their patterns/potential for dispersal, and their local and geographical distribution. The threats currently faced by coral reefs and the ongoing global effort to understand why corals are dying highlight the need to expand our understanding of basic coral physiology.

## Materials and Methods

**Figure 2:**
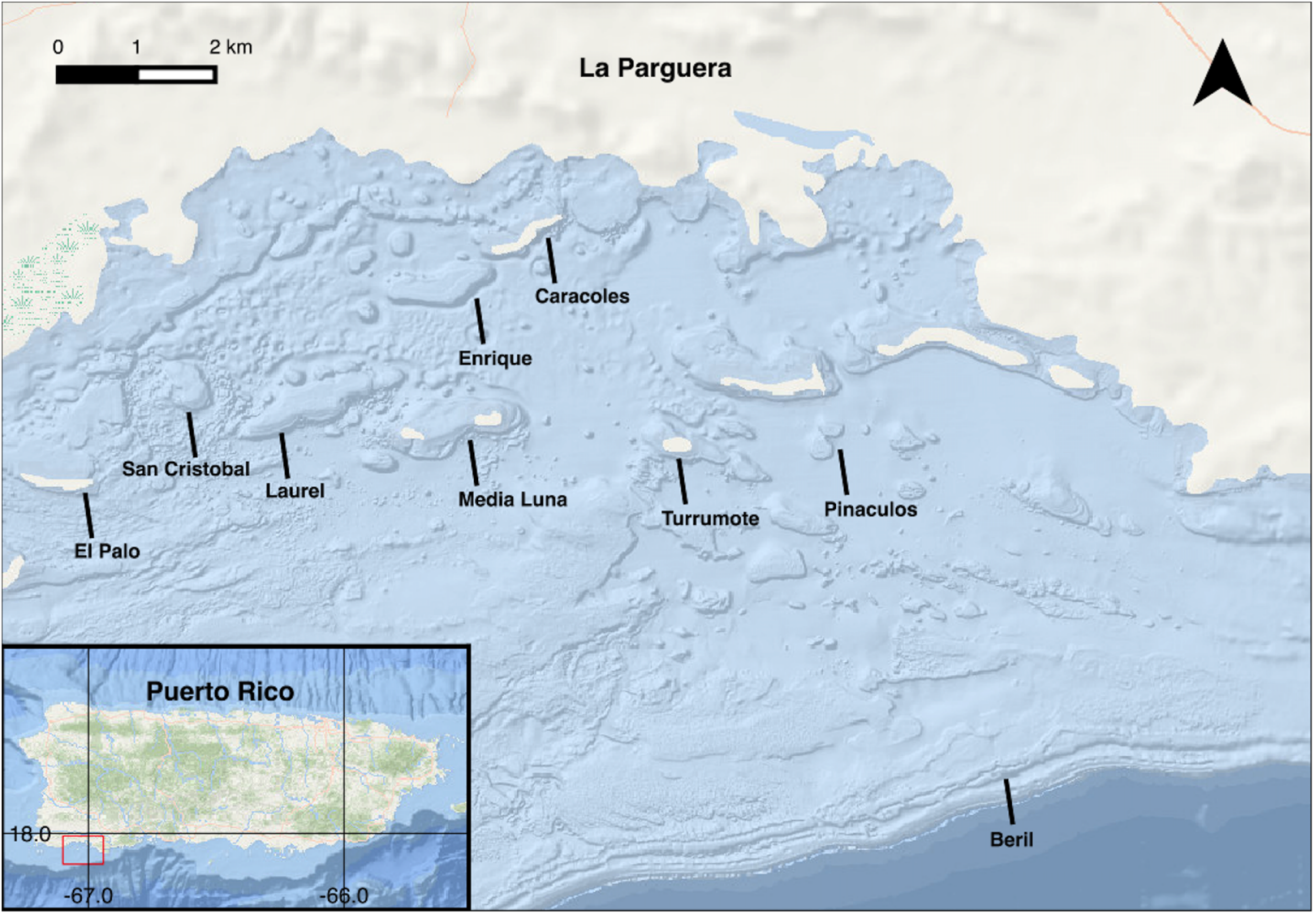
Map of La Parguera, Puerto Rico with study sites. Image made with QGIS using NOAA’s National Centers for Enviromental Information (NCEI) Multibeam Bathymetric Surveys Dataset.

Sampling for this study was carried out at La Parguera Natural Reserve, off the southwest coast of Puerto Rico (Fig. 2). This complex reef environment is among the many regions experiencing deterioration by anthropogenic and environmental climate influences at local and global scales. Coral reefs in La Parguera are important local economic drivers, supporting artisanal and recreational fishing, tourism, recreational activities and also protect coastal settlements, seagrass communities and other wetland habitats from the effects of hurricanes and coastal erosion (Ballantine et al., 2008).

**Figure 3:**
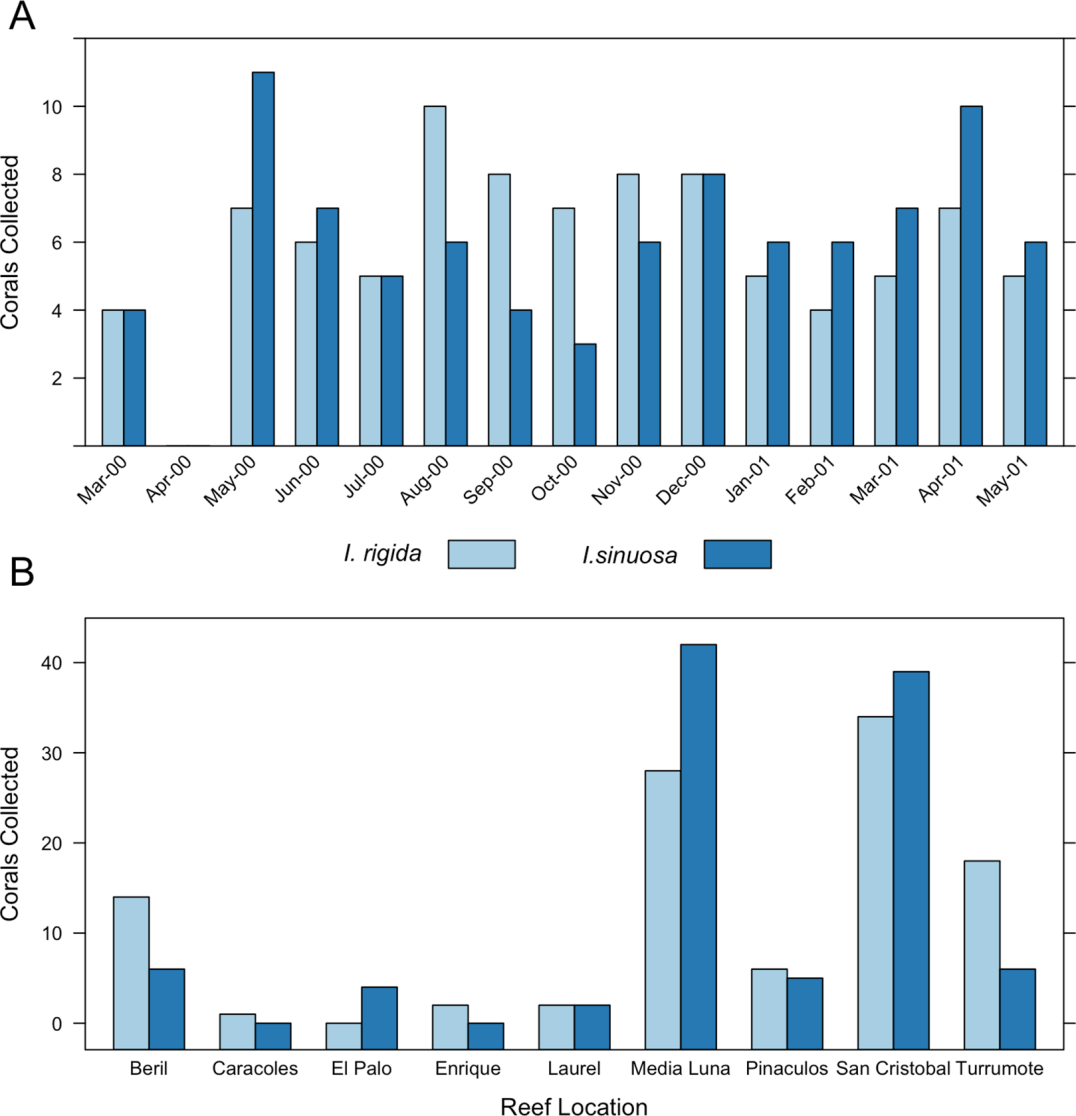
Collection data for [i]I. sinuosa[i] and [i]I. rigida[i]. (A) Number of samples collected per month, (B) Number of samples collected per location.

**Figure 4:**
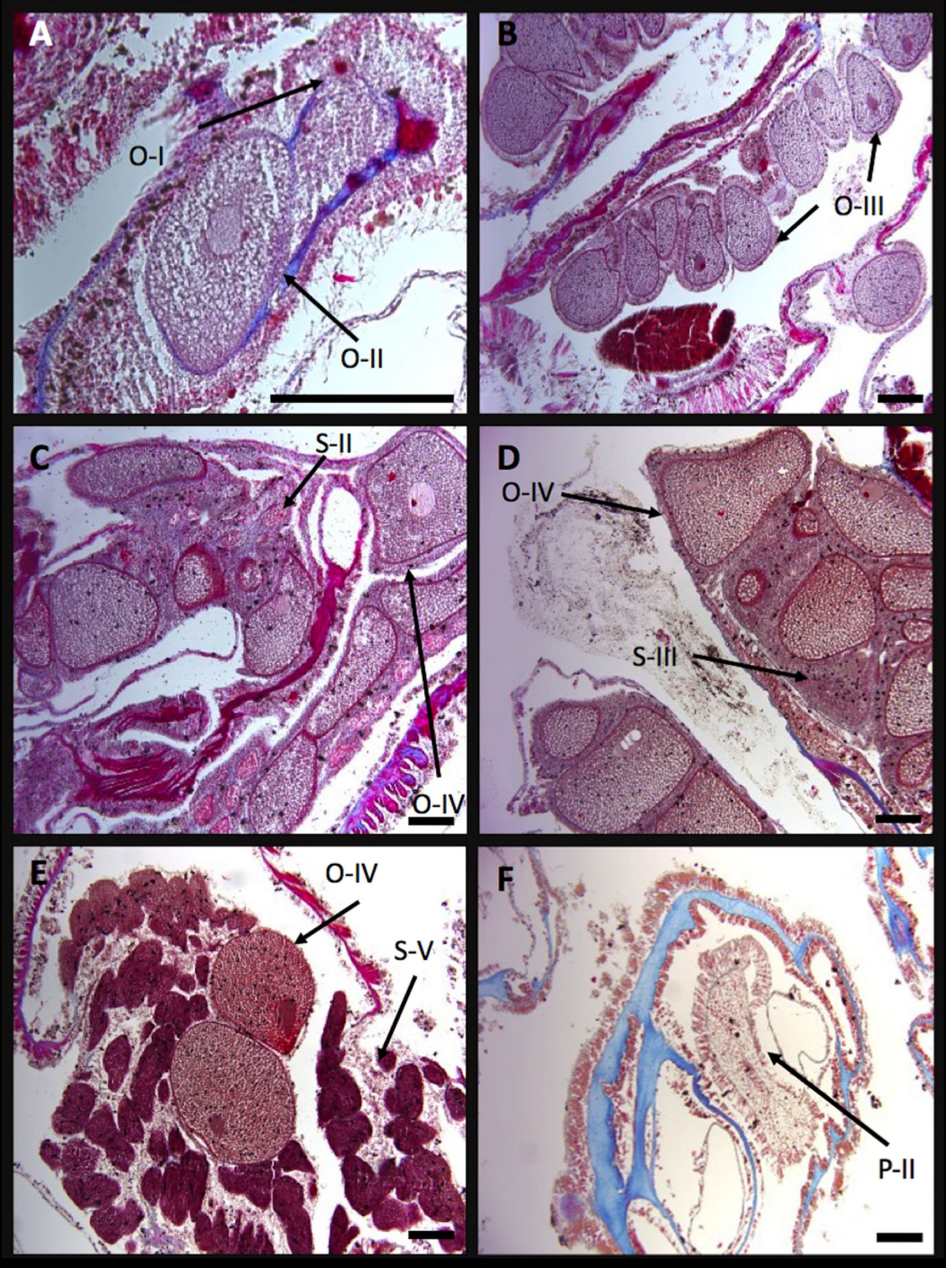
Developmental stages of oocytes (O) and spemaries (S) in *I. sinuosa*. (A) Stage I and II oocytes, (B) stage III oocytes, (C) stage II spermaries and stage IV oocytes, (D) stage IV oocytes and stage III spermaries, (E) stage IV oocytes and stage V spermaries, and (F) stage II planula. Scale bar measures 100 μm^2^.

At least five unique sample cores were collected monthly for 14 months between March 2000 and May 2001 (Fig. 3A). A total of 89 samples of each species were collected. Colonies were selected by searching in a zig-zag pattern over the distributional range of both species (5–18 m). Samples were collected from San Cristobal reef (17°55′24.88″N, 67°6′14.52″W), Caracoles reef (17°57′46.02″N, 67°2′8.21″W), Media Luna reef (17°56′22.68″N, 67°2′43.26″W), Pinaculos (17°56′1.13″N, 67°0′39.75″W), Turrumote reef (17°56′13.56″N, 67°1′8.92″W), Beril (17°52′47.85″N, 66°59′1.40″W), El Palo (17°55′50.2″N, 67°05′36.9″W), Laurel (17°55′50.2″N, 67°05′36.9″W) and Enrique (17°55′50.2″N, 67°05′36.9″W) (Figs. 3B and 4).

Sample cores were placed in Zenker Formalin (Helly’s solution) for 24 h, rinsed and then decalcified in 10% HCl solution. Tissues were then cleaned and placed in plastic tissue holders. Preserved samples were sequentially dehydrated in the rotary tissue processor under 70 and 95%, ethanol, Tissue Dry, and xylene solution (Tissue Clear III). Samples were embedded into Paraplast blocks then sectioned using a rotary microtome. The 8–10 strip sections (7–10 μm) were obtained from each embedded block and placed onto glass slides. Finished tissue slides were stained utilizing a modified Heidenhain’s Aniline-Blue method (Coolidge & Howard, 1979) to examine the maturation stages of gametocytes and embryos.

Slides were examined under an Olympus BX40 compound microscope coupled to an Olympus DP26 digital microscope camera. Images were captured utilizing Olympus cellSens 1.7 imaging software. The sexual pattern, gametogenic cycle and fecundity of each species were determined by observing the gametocyte development throughout the collection year. Gamete stages were characterized according to Szmant-Froelich, Reutter & Riggs (1985). Oocyte sizes were obtained using cellSens, by taking perpendicular measurements at the cell’s widest points. Cell length and width measurements were used to calculate geometric area. Fecundity was assessed by counting oocytes per mesentery (*I. sinuosa* n = 120; *I. rigida* n = 60) and per polyp (*I. sinuosa* n = 10; *I. rigida* n = 5) on histologic cross-sections during months with the highest proportion of mature oocytes (*I. sinuosa* April 2001 n = 5; *I. rigida* May 2001 n = 5).

In April 2012, several presumed gravid colonies of each species were collected and placed in an open seawater aquarium system to observe planulation. Two colonies of each species were placed within six-gallon aerated aquariums under continuously circulating seawater and daylight synchronized lights. Specimens were placed under mesh-lined PVC pipes allowing water to freely circulate. Traps were checked daily for larvae over a 90-day period.

### Statistical analyses

Results are expressed as means ± standard error. All statistical tests were performed using the RStudio 0.99.484 software platform (R Studio Team, 2015) using the stats package (R Development Core Team, 2015). Normality was assessed using the Shapiro-Wilk test performed with the R function shapiro.test. Equality of variance was tested using the F test performed with the R function var.test. Differences in fecundity were tested by means of a Wilcoxon rank sum test with continuity correction performed with the R function wilcoxon.test.

### Collection permit

All coral tissue samples were collected under a General Collection Permit granted by the Puerto Rico Department of Natural Resources (DNER) to the Faculty of the Department of Marine Sciences, University of Puerto Rico Mayaguez (UPRM).

## Results

### I. sinuosa

Stage I oocytes are small (78.92 ± 13.15 μm^2^), stain pink and are characterized by sparse cytoplasm and prominent nuclei (Fig. 4A). Oocytes originate within the linings of the mesoglea in the central regions of the mesenteries. Stage II oocytes are larger than stage I cells (144.54 ± 43.19 μm^2^), exhibit prominent nuclei and abundant cytoplasm (Fig. 4A). Stage III oocytes are larger than stage II (264.51 ± 37.24 μm^2^), tend to have a round shape, stain pink or red, and are characterized by many cytoplasmic globules which produce a grainy appearance (Fig. 4B). Stage IV oocytes are larger and boxier than stage III (376.69 ± 73.20 μm^2^). This stage is characterized by dark staining nuclei and large globules in the cytoplasm (Figs. 4C–4E).

No stage I spermaries were found, suggesting this stage occurs briefly and/or is difficult to differentiate using the current method. Stage II spermaries form small poorly defined bundles which form in the mesenteries surrounding oocytes (Fig. 4C). Stage III spermaries form small sacs with well-defined borders (Fig. 4D) and contain bright red staining spermatids. Stage IV spermaries stain dark red and are larger than stage III. Tails visible on spermatozoa at high magnification are indicative of stage V spermaries (Fig. 4E). Spermary sizes were not measured.

Stage I planulae are approximately the same size as stage IV oocytes (404.07 μm^2^) and stain pink. During this stage, zooxanthellae become visible within the planulae. Stage II planulae (455.45 ± 32.84 μm^2^) are characterized by an outer layer composed of columnar cells which contain nematocysts and cilia (Fig. 4F). Developing mesenteries can be seen within the gastrodermis of stage III planula (501.98 ± 44.68 μm^2^). Stage IV planula were not observed.

**Figure 5:**
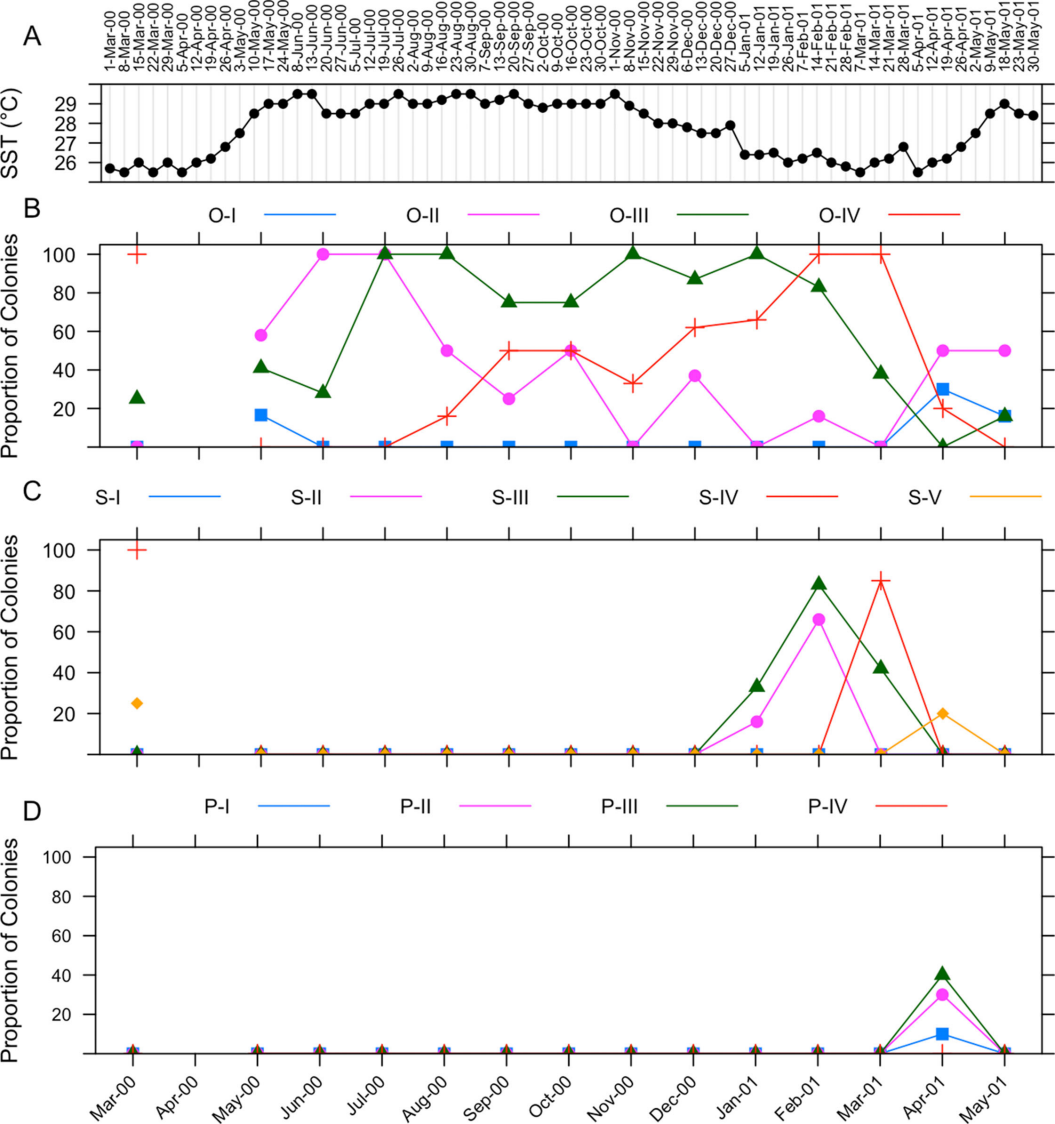
Gametogenic cycle of *I. sinuosa*. (A) Sea surface temperature ranges in La Parguera, Puerto Rico. Adjusted values of relative proportions of colonies of *I. sinuosa* in each gametogenetic stage of (B) oogenesis, (C) spermatogenesis, and (D) embryogenesis from March 2000 to May 2001. O-I, O-II, O-III, O-IV represent oocyte stages 1 through 4, respectively; S-I, S-II, S-III, S-IV, S-V represent spermary stages 1 through 5, respectively; P-I, P-II, P-III, P-IV represent planulae stages 1 through 4, respectively.

The gametogenic cycle of *I. sinuosa* is summarized in Fig. 5. Weekly sea surface temperature measurements taken during the collecting period are included for reference (Fig. 5A). Oogenesis in *I. sinuosa* lasts approximately 11 months (Fig. 5B). Onset of oogenesis was determined to occur during May 2000 and during April 2001. Onset of oogenesis was determined as the month of appearance of stage I and II oocytes after the culmination of the previous gametogenic cycle. Stage II oocytes were prevalent in tissues during all months sampled except during November 2000 and January 2001. Stage III oocytes were observed in all sampled months except April 2001. Stage IV oocytes were observed between August 2000 through May 2001.

Spermatogenesis takes places during four months (Fig. 5C). Onset of spermatogenesis was not determined because stage I spermaries were not identified. Stage II spermaries were observed during January through February 2001. Stage III spermaries were visible from January through March 2001. Stage IV spermaries were present in March 2001. Stage V spermaries were present in tissues in April 2001.

Stage I–III planulae were observed in histologic sections during April 2001 (Fig. 5D). The identification of planulae on tissue sections coincided with a sharp decrease in the proportion of colonies containing mature (IV) oocytes. No larvae were collected from specimens placed in aquaria for observation.

### I. rigida

**Figure 6:**
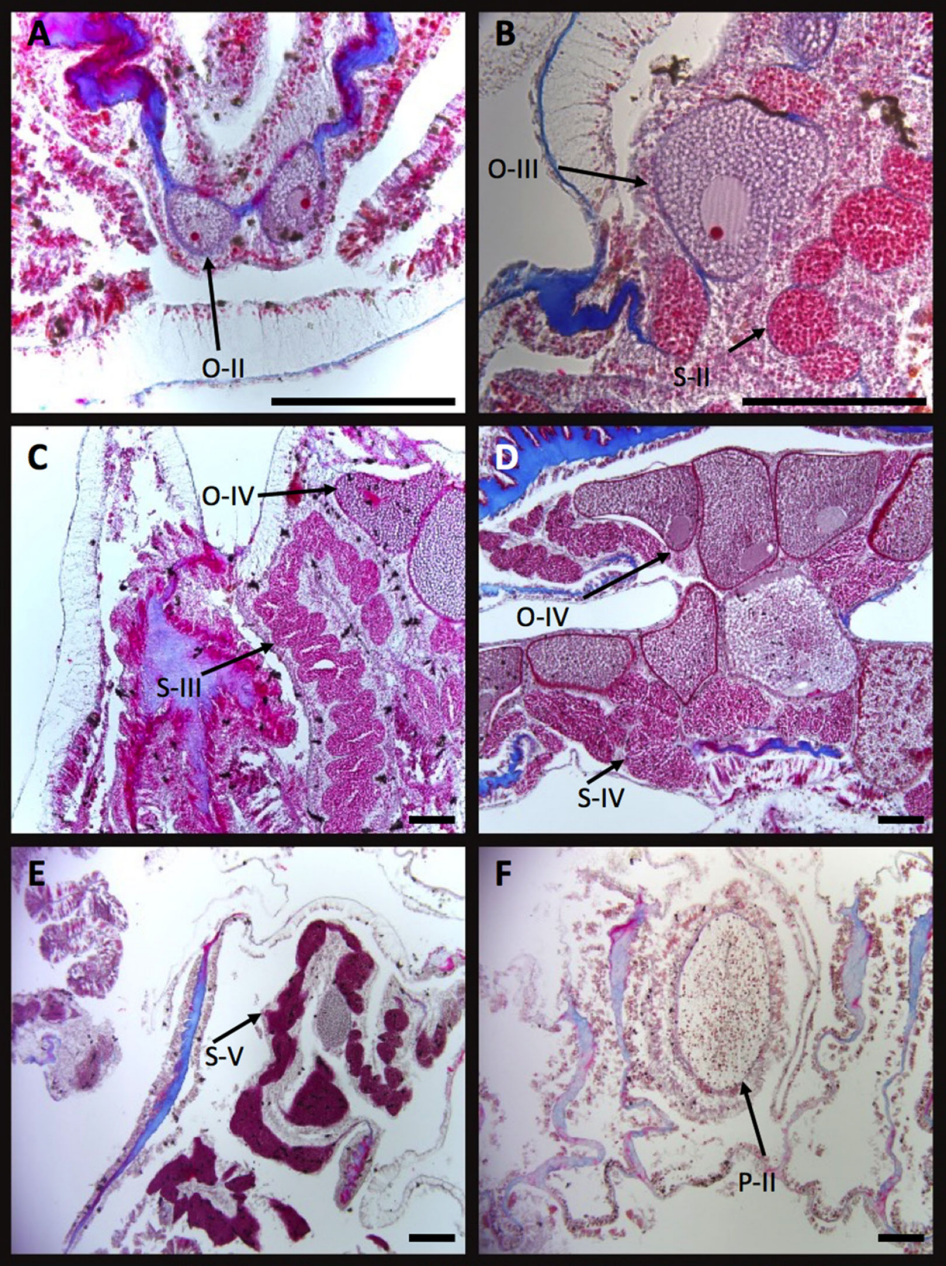
Developmental stages of oocytes (O) and spemaries (S) in *I. rigida*. (A) Stage II oocytes in the mesoglea, (B) stage III oocytes and Stage II spermaries, (C) stage III spermaries and stage IV oocytes, (D) stage IV oocytes and spermaries, (E) stage V spermaries, and (F) stage II planula. Scale bar measures 100 μm^2^.

Stage I oocytes are very small (72.97 ± 15.75 μm^2^) and are characterized by sparse cytoplasm and a large nucleus. Stage II oocytes are larger than stage I cells (101.25 ± 23.09 μm^2^), are ovoid shaped and feature a prominent nucleus and nucleolus (Fig. 6A). A pink-staining nucleus and red nucleolus can clearly be identified in many stage III oocytes (148.77 ± 49.35 μm^2^) (Fig. 6B). Stage IV oocytes are large (190.40 ± 45.18 μm^2^), irregularly shaped and contain large vacuoles in the ooplasma which give it a grainy appearance (Figs. 6C and 6D).

Stage I spermaries were not detected in *I. rigida*. Stage II spermaries were observed forming adjacent to stage III eggs (Fig. 6B). Spermaries typically adopt a spherical shape and often form in series resembling a string of beads (Figs. 6B and 6C). Stage III spermaries form small oblong sacs and stain red (Fig. 6C). Stage IV spermaries are densely packed with sperm, have irregular shapes, stain dark red to brown. Stage V spermaries stain darker than stage IV (Fig. 6E) but are characterized by tails on spermatozoa under high magnification. No measurements were collected for spermaries.

Stage I planulae are approximately the same size as stage IV oocytes (approximately 324.01 ± 71.64 μm^2^), stain pink, and contain zooxanthellae in the epidermis. Zooxanthellae were observed within planula beginning at this stage. Stage II planulae are larger (521.27 ± 84.18 μm^2^) (Fig. 6F) and exhibit an epidermis consisting of columnar epithelium similar to *I. sinuosa*. Stage III and stage IV larvae measure 818.91 ± 82.96 μm^2^ and 951.78 ± 176.36 μm^2^ respectively, and show clear development of the mesenteries.

**Figure 7:**
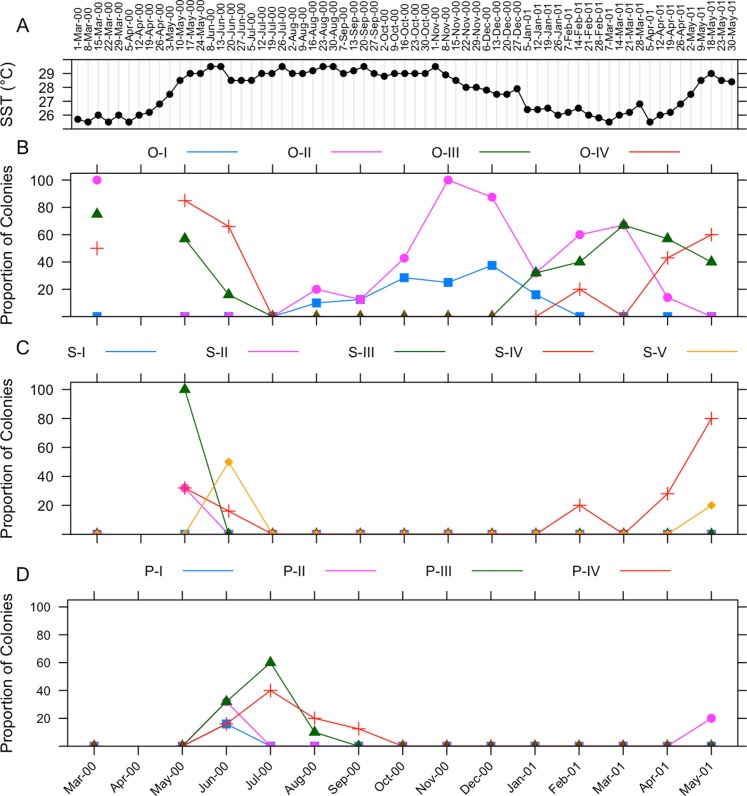
Gametogenic cycle of *I. rigida*. (A) Sea surface temperature ranges in La Parguera, Puerto Rico. Adjusted values of relative proportions of colonies of *I. sinuosa* in each gametogenetic stage of (B) oogenesis, (C) spermatogenesis, and (D) embryogenesis from March 2000 to May 2001. O-I, O-II, O-III, O-IV represent oocyte stages 1 through 4, respectively; S-I, S-II, S-III, S-IV, S-V represent spermary stages 1 through 5, respectively; P-I, P-II, P-III, P-IV represent planulae stages 1 through 4, respectively.

The gametogenic cycle of *I. rigida* is summarized in Fig. 7. Weekly sea surface temperature measurements taken during the collecting period are included for reference (Fig. 7A). Oogenesis in *I. rigida* lasts approximately 10 months (Fig. 7B). Oogenesis began during August 2000. Stage II oocytes were observed in tissues in March 2000 and August 2000 to April 2000. Stage III oocytes were observed in March 2000, May and June 2000 and from January 2001 through May 2001. Stage IV oocytes were observed in samples collected during March, May and June 2000, and February, April and May 2001.

Spermatogenesis in *I. rigida* is estimated to last approximately 2–3 months (Fig. 7C). Onset of spermatogenesis was not determined because stage I spermaries were not identified. Stage II spermaries were observed in May 2000. Stage III spermaries were visible in May 2000. Stage IV spermaries were observed first in June 2000. Stage V spermaries were observed in May 2000.

Stage I planulae were observed in June 2000 indicating the onset of embryogenesis (Fig. 7D). The appearance of planulae coincided with a sharp decrease in the proportion of colonies containing mature oocytes. Stage II planulae were observed during June 2000 and May 2001. Stage III planulae were observed from June through August 2000. Stage IV planulae were observed in tissues from June throughout September 2000. No larvae were collected from specimens placed in aquaria for observation.

### Fecundity

**Figure 8:**
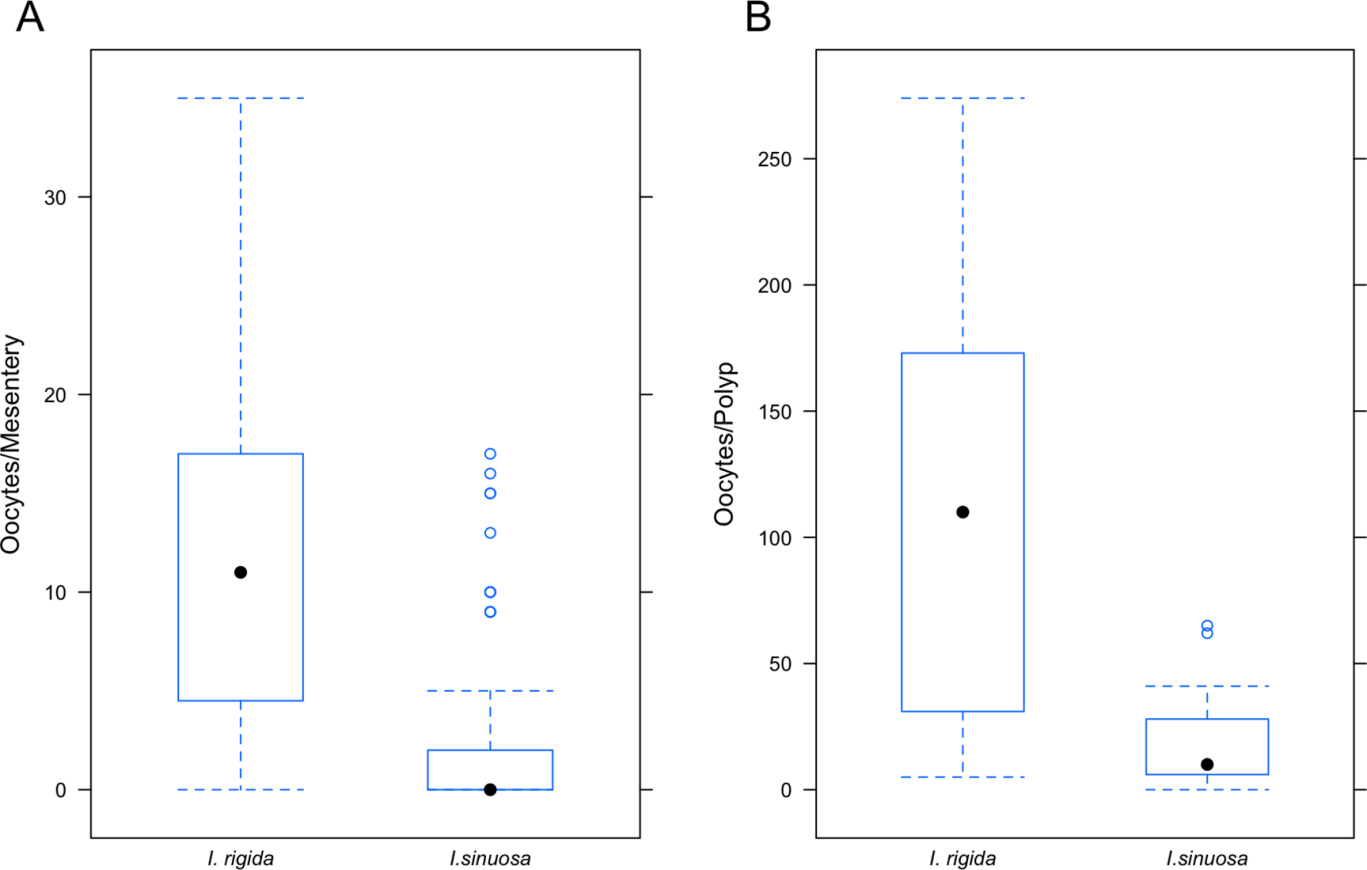
(A) Average mesenterial (eggs/mesentery) fecundity and (B) polyp (eggs/polyp) fecundity in *I. sinuosa* and *I. rigida*. Whiskers represent standard error.

Mesenterial fecundity in *I. sinuosa* (11.13 ± 8.27 oocytes/mesentery) was significantly higher (Wilcoxon-rank sum test, *W* = 1,208, *p* < 2.2 × 10^−16^) than in *I. rigida* (1.70 ± 3.52 oocytes/mesentery) (Fig. 8A). Polyp fecundity in *I. sinuosa* (110.11 ± 96.33 oocytes/polyp) was significantly higher (Wilcoxon-rank sum test, *W* = 18, *p* = 0.018) compared to *I. rigida* (20.45 ± 23.91 oocytes/polyp) (Fig. 8B).

### Oocyte size

**Figure 9:**
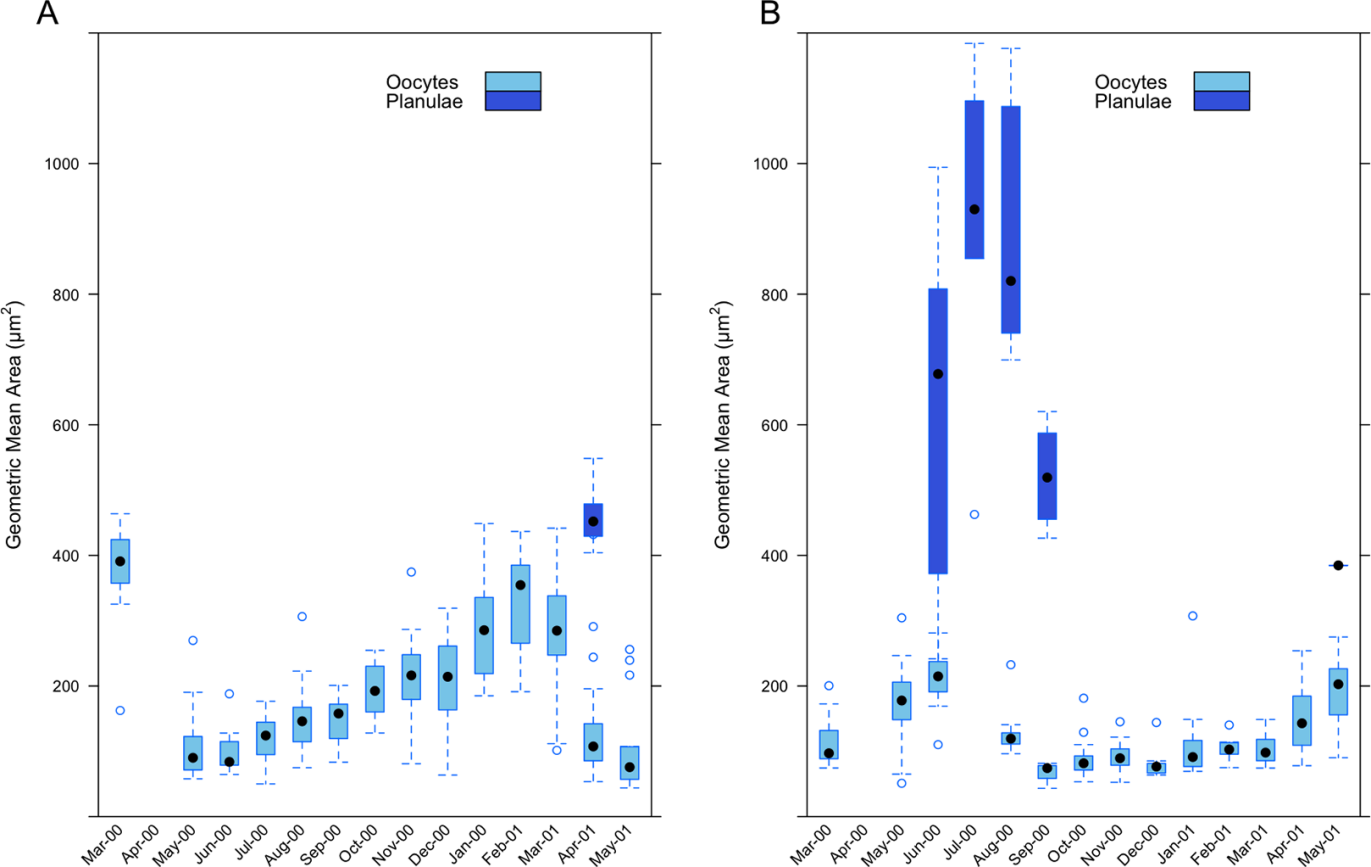
Monthly geometric mean oocyte and planulae areas in (A) *I. sinuosa* and (B) *I. rigida*.

Measurements of oocyte geometric area in *I. sinuosa* (range 43.94–463.79 μm^2^) show an increase in the size of oocytes as maturity progresses from April through March (Fig. 9A). Mean geometric area is lowest during the month of June 2000 (97.22 ± 28.85 μm^2^) and greatest during February 2001 (333.95 ± 74.32 μm^2^). The appearance of planulae in histological sections during the month of April 2001 (459.07 ± 45.83 μm^2^) (range: 404.07–548.49 μm^2^) coincides with a sharp decrease in mean geometric area of oocytes compared to the previous month (285.68 ± 96.46 vs. 143.28 ± 84.07 μm^2^). Measurements of oocyte geometric area in *I. rigida* (range 43.31–307.35 μm^2^) also show a trend of increasing oocyte size as maturity progresses from August through June (Fig. 9B). Mean geometric area is lowest during the month of September 2000 (68.35 ± 17.04 μm^2^) and greatest during June 2000 (210.54 ± 42.90 μm^2^). Mean planulae area was greatest during the month of July 2000 (909.48 ± 250.56 μm^2^) and ranged from 241.66–1,183.96 μm^2^. Mean oocyte geometric area was greater in *I. sinuosa* than in *I. rigida* (Wilcoxon-rank sum test, *W* = 43,911, *p* < 2.13 × 10^−13^); however, mean planulae geometric area was significantly higher in *I. rigida* compared to *I. sinuosa* (Wilcoxon-rank sum test, *W* = 186, *p* = 0.008).

## Discussion

Microscopic observations indicate that both *I. sinuosa* and *I. rigida* are simultaneous hermaphrodites (gametes of both sexes are present in a single individual at the same time). Gametes of both sexes are produced adjacent within the same mesentery (dygonism) in both species. Both species are brooders (bear live young) which transfer endosymbiotic zooxanthellae directly from parent to offspring. Both species are characterized by a single annual gametogenic cycle. This study represents the first description of the sexual characteristics of *I. rigida* and contradicts observations by Duerden (1902) which label *I. sinuosa* as a gonochoric species. The incorrect classification of *I. sinuosa* as the sole gonochoric outlier within the traditional Mussidae was a contrasting element in a group which is otherwise uniformly hermaphroditic (Duerden, 1902; Fadlallah, 1983; Richmond & Hunter, 1990). This study confirms the dominant pattern of sexual reproduction described for Mussid corals (Baird, Guest & Willis, 2009) and provides further support for conserved reproductive patterns within coral families (Harrison, 2011).

Traditional morphology-based classifications are being restructured by designating systematic affinities using molecular methods in combination with morphometric analyses. The traditional Mussidae family has recently undergone extensive restructuring by separating Indo-Pacific Mussids from their Atlantic counterparts which are more closely related to some members of the family Faviidae (Fukami et al., 2004; Fukami et al., 2008; Budd et al., 2012). The resulting ‘modern’ Mussidae (clade XXI) is composed of the genera *Mussa, Isophyllia, Mycetophyllia,* and *Scolymia* (Atlantic) under the Mussinae subfamily and *Favia* (Atlantic)*, Colpophyllia, Diploria, Pseudodiploria, Manicina* and *Mussismilia* under the Faviinae subfamily. Under the new classification, hermaphroditism has been exclusively documented within all genera of the subfamily Mussinae: *Mycetophillia* (Szmant-Froelich, 1985; Morales, 2006), *Scolymia* (Pires, Castro & Ratto, 2002; E. Weil, 2016, unpublished data) and *Mussa* (Steiner, 1993) and within the subfamily Faviinae: *Favia* (Soong, 1991)*, Colpophyllia* (E. Weil, 2016, unpublished data)*, Diploria* (Weil & Vargas, 2010) *Pseudodiploria* (Weil & Vargas, 2010)*, Manicina* (Johnson, 1992)*, Mussismilia* (Pires, Castro & Ratto, 1999) (Table 1). Mode of development within the modern Mussidae is mixed; both brooding and spawning species are present. Brooding has been documented within *Mycetophyllia* (Morales, 2006), *Scolymia* (Pires, Castro & Ratto (2002); E. Weil, 2016, unpublished data), and *Manicina* (Johnson, 1992). Broadcast spawning occurs in *Colpophyllia* (E. Weil, 2016, unpublished data), *Diploria* (Weil & Vargas, 2010), *Pseudodiploria* (Weil & Vargas, 2010), and *Favia* (Soong, 1991). Sexual mode exhibits more plasticity than sexuality (Van Moorsel, 1983; Harrison, 1985): contrasting modes of development exist within families and even within genera (Harrison, 2011).

**Table 1:** Comparison of reproductive characteristics of *Mussidae* (Clade XXI).

Subfamily	Genus	Species	Sexual pattern	Mode of development	Source
Mussinae	*Mussa*	*M. angulosa*	H		Steiner (1993)
	*Isophyllia*	*I. rigida*	**H**	**Brooding**	**This study**
		*I. sinuosa*	**H**	**Brooding**	Duerden (1902) and **This study**
	*Mycetophyllia*	*M. ferox*	H	Brooding	Szmant-Froelich (1984), Szmant (1986) and Morales (2006)
		*M. aliciae*	H	Brooding	Morales (2006)
		*M. lamarckiana*	H	Brooding	Morales (2006)
		*M. danaana*	H	Brooding	Morales (2006)
		*M. reesi*			
	*Scolymia* (Atlantic)	*S. cubensis*	H	Brooding	E. Weil (2016, unpublished data)
		*S. lacera*	H	Brooding	E. Weil (2016, unpublished data)
		*S. wellsi*	H	Brooding	Pires, Castro & Ratto (2002)
Faviinae	*Favia* (Atlantic)	*F. fragrum*	H	Broadcast	Duerden (1902), Fadlallah (1983), Szmant (1986), Richmond & Hunter (1990) and Soong (1991)
	*Colpophyllia*	*C. amaranthus*	H	Broadcast	E. Weil (2016, unpublished data)
		*C. natans*	H	Broadcast	Steiner (1995), Hagman, Gittings & Deslarzes (1998), Boland (1998) and E. Weil (2016, unpublished data)
	*Diploria*	*D.labyrinthiformis*	H	Broadcast	Duerden (1902), Fadlallah (1983), Wyers, Barnes & Smith (1991) and Weil & Vargas (2010)
	*Pseudodiploria*	*D. clivosa*	H	Broadcast	Soong (1991) and Weil & Vargas (2010)
		*D. strigosa*	H	Broadcast	Szmant (1986), Richmond & Hunter (1990), Soong, 1991, Steiner (1995) and Weil & Vargas (2010)
	*Manicina*	*M. areolata*	H	Brooding	Duerden (1902), Fadlallah (1983), Richmond & Hunter (1990) and Johnson (1992)
	*Mussismilia*	*M.hispida*	H	Broadcast	Neves & Pires (2002) and Pires, Castro & Ratto (1999)
		*M. hartii*	H	Broadcast	Pires, Castro & Ratto (1999)
		*M. brazilensis*	H	Broadcast	Pires, Castro & Ratto (1999)

**Note:**

*H*, hermaphroditic; *G*, gonochoric.

Szmant (1986) suggested that sexual mode is potentially a function of habitat stability, where successful recruiters would be small, rapidly maturing species, which produce many offspring over short periods but subject to high mortality rates. Thus, the sexual modality of species occupying unstable habitats would gravitate towards brooding because it increases the chances of successful recruitment by reducing gamete and larval mortality even in low population densities. Edinger & Risk (1995) on noting a correlation between brooding and eurytopy, hypothesized that brooding corals may preferentially survive in unstable habitats due to higher recruitment success. The benefits provided by the brooding modality may partially explain why, in recent decades, brooding corals have begun to dominate some Caribbean reefs following degradation by natural and anthropogenic disturbances (Hughes, 1994; Mumby, 1999; Knowlton, 2001; Irizarry-Soto & Weil, 2009).

The close proximity of oocytes and spermaries within the same mesentery (dygonism) in *I. sinuosa* and *I. rigida* suggests that it is possible that self-fertilization can occur in these species. Generally, self-fertilization is not a favored method of fertilization in corals due to possibility of inbreeding depression (Knowlton & Jackson, 1993). Selfing, however, is thought to be advantageous in sessile hermaphrodites which are ecologically distant from other mates and may have limited access to gametes of the other sex, providing a viable alternative for successful fertilization (Ayre & Miller, 2004; Darling et al., 2012; Sawada, Morita & Iwano, 2014). These corals may then switch to sexually produced larvae as population sizes increase (Ayre & Resing, 1986). Selfing has been documented in the brooding corals *Seriatopora hystrix* (Sherman, 2008)*, Favia fragum* and *Porites astreoides* (Brazeau, Gleason & Morgan, 1998).

The duration of the gametogenic cycle is similar in *I. sinuosa* and *I. rigida* (11 and 10 months, respectively). Long oocyte generation times, differential gamete maturation, and long brood retention times in *Isophyllia* suggest the possibility of multiple brooding events during a single gametogenetic cycle. This strategy may increase reproductive output due to space limitations within polyps. A single annual gametogenetic cycle is the dominant pattern in most broadcasting corals such as *Orbicella, Montastraea*, *Diploria*, *Porites*, *Acropora*, *Siderastrea* (Szmant, 1986; (Vargas-Ángel & Thomas, 2002; Weil & Vargas, 2010) and brooding Caribbean corals like *Porites* and *Mycetophyllia* (Szmant, 1986; Soong, 1993; Vermeij et al., 2004; Morales, 2006). Multiple spawning events have been documented in *Acanthastrea lordhowensis* (Wilson & Harrison, 1997) and cannot be ruled out in these species.

Both species differ in the timing of oogenesis and planulogenesis events by various months which suggests that opportunities for hybridization between both species are limited. The dates of onset of oogenesis in both species (May in *I. sinuosa* and August in *I. rigida*) coincide with warm local sea surface temperatures suggesting seasonal synchronization of the gametogenic cycle. In *I. sinuosa*, planulae were observed in histologic sections during April 2001 which suggests that fertilization occurred during early April (most recent Full Moon: April 9). In *I. rigida*, planulae were observed in June 2000 which suggests a fertilization date in late May (most recent Full Moon: May 6, 2001). Various environmental factors have been shown to correlate with coral reproductive cycles and may play a role in their synchronization, including sea temperature, salinity, day length, light/dark cycles and tidal cycles (Harrison & Wallace, 1990). Van Woesik, Lacharmoise & Köksal (2006) showed experimentally that some coral spawning schedules correlate strongly with solar insolation levels prior to gamete release, however, water temperatures are highly influential in determining actual gamete maturity. van Woesik (2009) also demonstrated a positive correlation between the duration of regional wind calm periods and the coupling of mass coral spawnings. Studies with the brooding coral *Pocillopora damicornis* revealed that synchronization of larval production was lost under constant artificial new moon and full moon conditions, demonstrating that planulation in some species is linked to nighttime irradiance (Jokiel, Ito & Liu, 1985).

Acquisition of the endosymbiont *Symbiodinium* in *Isophyllia occurs* directly from parent to offspring (vertical transmission), a characteristic strongly linked to the brooding modality (Baird, Guest & Willis, 2009). Vertical symbiont transmission may be advantageous by providing larvae with various *Symbiodinium* genotypes which may improve their ability to recruit successfully and grow in different environmental conditions (Padilla-Gamiño et al., 2012). Brooded larvae are capable of motility immediately or shortly after planulation (Fadlallah, 1983), in contrast to broadcast spawned propagules which are positively buoyant and may take between 12–72 h to become motile (Baird, Guest & Willis, 2009). By avoiding the surface, brooded larvae may better avoid exposure to high levels of solar radiation which may overwhelm the photosynthetic capacities of zooxanthellae producing oxygen radicals (Tchernov et al., 2004) and cause tissue damage and mortality (Lesser et al., 1990). However, under high temperature conditions, larvae of corals with vertical symbiont transmission may suffer higher oxidative stress and tissue damage, suggesting that these corals may be more vulnerable to the effects of ocean warming (Yakovleva et al., 2009).

There is increasing evidence that sexual reproduction in corals is highly susceptible to natural and anthropogenic stressors that reduce fecundity, fertilization success, and larval survival (Harrison & Wallace, 1990; Harrison, 2011). Increases in sea surface temperatures as a consequence of global warming have produced widespread coral bleaching events and disease outbreaks with massive mortality of susceptible individuals. This worldwide decline of coral reefs underscores the need for understanding sexual reproduction in corals as the only mechanism capable of safeguarding their future. Sexual recombination is an important prerequisite for the selection of individuals which are to be able to adapt to the pressures of a changing environment. A greater understanding of the mechanisms and variables in sexual reproduction in corals, in combination with knowledge of the taxonomy and variability of the species, is essential for any coral reef management strategy (Harrison & Wallace, 1990).

## Supplemental Information

10.7717/peerj.2665/supp-1 Supplemental Information 1 Dataset consisting of raw oocyte, spermary and planula prevalence, oocyte sizes and fecundity obtained from histologic sections of *I. sinuosa* and *I. rigida*.

## Additional Information and Declarations

### Field Study Permissions

The following information was supplied relating to field study approvals (i.e., approving body and any reference numbers): Corals were sampled under a General Collection Permit granted by the Puerto Rico Department of Natural Resources (DNER) to the Faculty of the Department of Marine Sciences UPRM.

### Data Deposition

The following information was supplied regarding data availability: The raw data has been supplied as Supplemental Dataset Files.

## References

[ref-1] Ayre DJ, Miller KJ (2004). Where do clonal coral larvae go? Adult genotypic diversity conflicts with reproductive effort in the brooding coral, *Pocillopora damicornis*. Marine Ecology Progress Series.

[ref-2] Ayre DJ, Resing JM (1986). Sexual and asexual production of planulae in reef corals. Marine Biology.

[ref-3] Babcock RC, Bull GD, Harrison PL, Heyward AJ, Oliver JK, Wallace CC, Willis BL (1986). Synchronous spawnings of 105 scleractinian coral species on the Great Barrier Reef. Marine Biology.

[ref-4] Baird AH, Guest JR, Willis BL (2009). Systematic and biogeographical patterns in the reproductive biology of scleractinian corals. Annual Review of Ecology, Evolution, and Systematics.

[ref-5] Ballantine DL, Appeldoorn RS, Yoshioka P, Weil E, Armstrong R, Garcia JR, Otero E, Pagan F, Sherman C, Hernandez-Delgado EA, Bruckner A, Lilyestrom C, Riegl B, Dodge RE (2008). Biology and ecology of Puerto Rican coral reefs. Coral Reefs of the USA.

[ref-6] Boland GS (1998). Spawning observations of the scleractinian coral *Colpophyllia natans* in the northwest Gulf of Mexico. Gulf of Mexico Science.

[ref-7] Brazeau DA, Gleason DF, Morgan ME (1998). Self-fertilization in brooding hermaphroditic Caribbean corals: evidence from molecular markers. Journal of Experimental Marine Biology and Ecology.

[ref-8] Budd AF, Fukami H, Smith ND, Knowlton N (2012). Taxonomic classification of the reef coral family Mussidae (Cnidaria: Anthozoa: Scleractinia). Zoological Journal of the Linnean Society.

[ref-9] Coolidge BJ, Howard RM (1979). Animal Histology Procedures.

[ref-10] Darling ES, Alvarez-Filip L, Oliver TA, McClanahan TR, Côté IM (2012). Evaluating life-history strategies of reef corals from species traits. Ecology Letters.

[ref-11] Duerden JE (1902). West Indian Madreporarian Polyps.

[ref-75] Edinger EN, Risk MJ (1995). Preferential survivorship of brooding corals in a regional extinction. Paleobiology.

[ref-12] Edmunds PJ (2005). Effect of elevated temperature on aerobic respiration of coral recruits. Marine Biology.

[ref-13] Fadlallah YH (1983). Sexual reproduction, development and larval biology in scleractinian corals. Coral Reefs.

[ref-14] Fukami H, Budd AF, Paulay G, Solé-Cava A, Chen CA, Iwao K, Knowlton N (2004). Conventional taxonomy obscures deep divergence between Pacific and Atlantic corals. Nature.

[ref-15] Fukami H, Chen CA, Budd AF, Collins A, Wallace C, Chuang Y-Y, Chen C, Dai C-F, Iwao K, Sheppard C, Knowlton N (2008). Mitochondrial and nuclear genes suggest that stony corals are monophyletic but most families of stony corals are not (Order Scleractinia, Class Anthozoa, Phylum Cnidaria). PLoS ONE.

[ref-16] Hagman DK, Gittings SR, Deslarzes KJP (1998). Timing, species participation, and environmental factors influencing annual mass spawning at the Flower Garden Banks (Northwest Gulf of Mexico). Gulf of Mexico Science.

[ref-17] Harrison PL, Gabrie C, Salvat B (1985). Sexual characteristics of scleractinian corals: systematic and evolutionary implications.

[ref-18] Harrison PL, Dubinsky Z, Stambler N (2011). Sexual reproduction of scleractinian corals. Coral Reefs: An Ecosystem in Transition.

[ref-19] Harrison PL, Wallace CC, Dubinsky Z (1990). Reproduction, dispersal and recruitment of scleractinian corals. Coral Reefs, Ecosystems of the World.

[ref-22] Hughes TP (1994). Catastrophes, phase shifts, and large-scale degradation of a Caribbean coral reef. Science-AAAS-Weekly Paper Edition.

[ref-23] Irizarry-Soto E, Weil E (2009). Spatial and temporal variability in juvenile coral densities, survivorship and recruitment in La Parguera, southwestern Puerto Rico. Caribbean Journal of Science.

[ref-24] Johnson KG (1992). Synchronous planulation of *Manicina areolata* (Scleractinia) with lunar periodicity. Marine Ecology Progress Series.

[ref-25] Jokiel PL, Ito RY, Liu PM (1985). Night irradiance and synchronization of lunar release of planula larvae in the reef coral *Pocillopora damicornis*. Marine Biology.

[ref-26] Knowlton N (2001). The future of coral reefs. Proceedings of the National Academy of Sciences of the United States of America.

[ref-27] Knowlton N, Jackson JB (1993). Inbreeding and outbreeding in marine invertebrates. The Natural History of Inbreeding and Outbreeding.

[ref-28] Lesser MP, Stochaj WR, Tapley DW, Shick JM (1990). Bleaching in coral reef anthozoans: effects of irradiance, ultraviolet radiation, and temperature on the activities of protective enzymes against active oxygen. Coral Reefs.

[ref-29] Metz JAJ, Nisbet RM, Geritz SAH (1992). How should we define ‘fitness’ for general ecological scenarios?. Trends in Ecology & Evolution.

[ref-30] Morales JA (2006). Sexual reproduction in the Caribbean coral genus *Mycetophyllia*, in La Parguera Puerto Rico.

[ref-31] Mumby PJ (1999). Bleaching and hurricane disturbances to populations of coral recruits in Belize. Marine Ecology Progress Series.

[ref-32] Neves E, Pires D (2002). Sexual reproduction of Brazilian coral *Mussismilia hispida* (Verrill, 1902). Coral Reefs.

[ref-33] Padilla-Gamiño JL, Pochon X, Bird C, Concepcion GT, Gates RD (2012). From parent to gamete: vertical transmission of *Symbiodinium* (Dinophyceae) ITS2 sequence assemblages in the reef building coral *Montipora capitata*. PLoS ONE.

[ref-35] Pinzon J, Weil E (2011). Cryptic species in the Atlantic-Caribbean scleractinian genus *Meandrina*: a multi-variable review of the taxonomy and description of the new species *Meandrina jacksoni*. Bulletin of Marine Science.

[ref-36] Pires DO, Castro CB, Ratto CC (1999). Reef coral reproduction in the Abrolhos Reef Complex, Brazil: the endemic genus *Mussismilia*. Marine Biology.

[ref-37] Pires DO, Castro CB, Ratto CC, Moosa MK, Soemodihardjo S, Soegiarto A, Romimohtarto K, Nontji, A, Nontji A, Soekarnoand, Suharsono (2002). Reproduction of the solitary coral Scolymia wellsi Laborel (Cnidaria, Scleractinia) from the Abrolhos reef complex, Brazil.

[ref-38] R Development Core Team (2015). R: A Language and Environment for Statistical Computing.

[ref-39] R Studio Team (2015). RStudio: Integrated Development for R.

[ref-40] Richmond RH, Hunter CL (1990). Reproduction and recruitment of corals: comparisons among the Caribbean, the Tropical Pacific, and the Red Sea. Marine Ecology Progress Series.

[ref-42] Sawada H, Morita M, Iwano M (2014). Self/non-self recognition mechanisms in sexual reproduction: new insight into the self-incompatibility system shared by flowering plants and hermaphroditic animals. Biochemical and Biophysical Research Communications.

[ref-43] Sherman CDH (2008). Mating system variation in the hermaphroditic brooding coral, *Seriatopora hystrix*. Heredity.

[ref-44] Soong K (1991). Sexual reproductive patterns of shallow-water reef corals in Panama. Bulletin of Marine Science.

[ref-45] Soong K (1993). Colony size as a species character in massive reef corals. Coral Reefs.

[ref-46] Steiner SCC (1993). Comparative ultrastructural studies on scleractinian spermatozoa (Cnidaria, Anthozoa). Zoomorphology.

[ref-47] Steiner SCC (1995). Spawning in scleractinian corals from SW Puerto Rico (West Indies). Bulletin of Marine Science.

[ref-72] Szmant-Froelich A, Reutter M, Riggs L (1985). Sexual reproduction of *Favia fragum* (Esper): lunar patterns of gametogenesis, embryogenesis and planulation in Puerto Rico. Bulletin of Marine Science.

[ref-48] Szmant AM (1986). Reproductive ecology of Caribbean reef corals. Coral Reefs.

[ref-50] Szmant-Froelich A (1984). Reef coral reproduction: diversity and community patterns.

[ref-51] Szmant-Froelich A, Gabrie C, Salvat B (1985). The effect of colony size on the reproductive ability of the Caribbean coral *Montastrea annularis* (Ellis and Solander).

[ref-52] Tchernov D, Gorbunov MY, de Vargas C, Yadav SN, Milligan AJ, Häggblom M, Falkowski PG (2004). Membrane lipids of symbiotic algae are diagnostic of sensitivity to thermal bleaching in corals. Proceedings of the National Academy of Sciences of the United States of America.

[ref-53] Van Moorsel GWNM (1983). Reproductive strategies in two closely related stony corals (*Agaricia*, Scleractinia). Marine Ecology Progress Series.

[ref-54] van Woesik R (2009). Calm before the spawn: global coral spawning patterns are explained by regional wind fields. Proceedings of the Royal Society of London B: Biological Sciences.

[ref-55] Van Woesik R, Lacharmoise F, Köksal S (2006). Annual cycles of solar insolation predict spawning times of Caribbean corals. Ecology Letters.

[ref-74] Vargas-Ángel B, Thomas J (2002). Sexual reproduction of *Acropora cervicornis* in nearshore waters off Fort Lauderdale, Florida, USA. Coral Reefs.

[ref-56] Vermeij MJA (2006). Early life-history dynamics of Caribbean coral species on artificial substratum: the importance of competition, growth and variation in life-history strategy. Coral Reefs.

[ref-57] Vermeij MJA, Sampayo E, Bröker K, Bak RPM (2004). The reproductive biology of closely related coral species: gametogenesis in *Madracis* from the southern Caribbean. Coral Reefs.

[ref-59] Weil E (2003). The corals and coral reefs of Venezuela. Latin American Coral Reefs.

[ref-61] Weil E, Croquer A, Urreiztieta I (2009). Temporal variability and impact of coral diseases and bleaching in La Parguera, Puerto Rico from 2003–2007. Caribbean Journal of Science.

[ref-66] Weil E, Vargas WL (2010). Comparative aspects of sexual reproduction in the Caribbean coral genus *Diploria* (Scleractinia: Faviidae). Marine Biology.

[ref-68] Wilson JR, Harrison PL, Lessios H.A , Macintyre IG (1997). Sexual reproduction in high latitude coral communities at the Solitary Islands, eastern Australia.

[ref-69] Wyers SC, Barnes HS, Smith SR (1991). Spawning of hermatypic corals in Bermuda: a pilot study. Hydrobiologia.

[ref-70] Yakovleva IM, Baird AH, Yamamoto HH, Bhagooli R, Nonaka M, Hidaka M (2009). Algal symbionts increase oxidative damage and death in coral larvae at high temperatures. Marine Ecology Progress Series.

